# Correction: UO_2_^2+^-mediated ring contraction of pyrihexaphyrin: synthesis of a contracted expanded porphyrin-uranyl complex

**DOI:** 10.1039/c9sc90138h

**Published:** 2019-07-09

**Authors:** James T. Brewster, Harrison D. Root, Daniel Mangel, Adam Samia, Hadiqa Zafar, Adam C. Sedgwick, Vincent M. Lynch, Jonathan L. Sessler

**Affiliations:** a Department of Chemistry , The University of Texas at Austin , 105 East 24th St., Stop A5300 , Austin , Texas 78712 , USA . Email: sessler@cm.utexas.edu

## Abstract

Correction for ‘UO_2_^2+^-mediated ring contraction of pyrihexaphyrin: synthesis of a contracted expanded porphyrin-uranyl complex’ by James T. Brewster *et al.*, *Chem. Sci.*, 2019, **10**, 5596–5602.



## 

We regret that the CCDC number 1893382 was given incorrectly in the original manuscript. The correct CCDC number is 1893882. The crystal structure data file for the original manuscript has been updated to reflect this change.

The Royal Society of Chemistry apologises for these errors and any consequent inconvenience to authors and readers.

